# Reliability and Sensitivity of Enode/Vmaxpro Sensor for Muscle Power Assessment

**DOI:** 10.3390/life14121706

**Published:** 2024-12-23

**Authors:** Milan Marković, Lazar Toskić, Veroljub Stanković, Radenko Arsenijević, Nikola Aksović, Bojan Bjelica, Saša Bubanj, Tatiana Dobrescu

**Affiliations:** 1 Faculty of Sport and Physical Education, University of Priština-Kosovska Mitrovica, 38218 Leposavic, Serbia; milan.markovic@pr.ac.rs (M.M.); lazar.toskic@pr.ac.rs (L.T.); veroljub.stankovic@pr.ac.rs (V.S.); radenko.arsenijevic@pr.ac.rs (R.A.); nikola.aksovic@pr.ac.rs (N.A.); 2 Faculty of Sport, University “Union—Nikola Tesla”, 11070 Belgrade, Serbia; 3 Faculty of Physical Education and Sports, University of East Sarajevo, 71126 Lukavica, Bosnia and Herzegovina; vipbjelica@gmail.com; 4 Faculty of Sport and Physical Education, University of Nis, 18000 Nis, Serbia; 5 Department of Physical Education and Sport Performance, Vasile Alecsandri University, 600115 Bacau, Romania; tatiana.dobrescu@ub.ro

**Keywords:** biomechanics, diagnostics, power, velocity, isoinertial dynamometry

## Abstract

(1) Background: Regardless of the level of physical activity, performance monitoring is a valuable component of the training process. The aim of this research was to assess the reliability and sensitivity of parameter measurements using the Enode/Vmaxpro sensor. (2) Methods: Metric characteristics were examined for average velocity, peak velocity, average power, peak power, movement length, and movement duration. Twenty-seven participants (15 men and 12 women) underwent body composition analysis and testing on a combined leg extension/leg curl machine, performing the exercises with each leg individually under a 30% body mass load. Descriptive statistics, reliability analyses, and difference analyses were conducted to evaluate repeatability and sensitivity levels. The significance threshold was set at the level 0.05. (3) Results: Reliability parameters were found to be statistically significant, both overall (ICC: 0.937–0.991) and separately for men (ICC: 0.899–0.984) and women (ICC: 0.908–0.990). Sensitivity was confirmed through significant differences based on sex (*p* = 0.000), activity level (*p* = 0.000), and movement type (*p* = 0.000). No statistically significant differences were observed between right and left leg performance. (4) Conclusions: The findings suggest that the Enode/Vmaxpro sensor demonstrates sufficient sensitivity and reliability for muscle power testing in biomechanics and sports diagnostics.

## 1. Introduction

Every physical activity, whether recreational or professional, includes resistance training (RT) as a primary or secondary component of the training process [1]. Regardless of the level of engagement in physical activity, performance monitoring from the perspective of muscle power is a desirable part of the entire training process, both for tracking training effects and for motivational purposes [2,3].

According to Cormie, McGuigan, and Newton [4], muscles possess a wide range of mechanical properties that influence power output. Since power is the product of force and velocity, the force/velocity (F-V) relationship plays a key role in generating maximum power output. For this reason, all testing methods—ranging from three-dimensional imaging to linear encoders and newer methods—are based on monitoring the load and its movement speed [5]. The influence of technological advancements in sports diagnostics has contributed to the development of equipment (hardware/software solutions) for testing and monitoring both physical abilities and sports performance levels [6].

Although all testing methods are based on speed measurement, from which other parameters are derived, factors such as space requirements, time and method of data processing, application versatility, and financial affordability must be considered, as they can significantly impact their practical use. Additionally, the applicability of these methods depends on the metric characteristics of the test and the equipment used, specifically validity, reliability, and sensitivity.

The increasing demand for simpler and more affordable solutions for testing the physical abilities of both professional and recreational athletes has spurred the development of numerous sensors. Thanks to technological advances, the scope of testing outside the laboratory has expanded significantly. Accelerometers, often integrated with global positioning systems, are among these innovations. Accelerometers are typically accompanied by a gyroscope and a gravimeter, forming an inertial measurement unit (IMU), which offers advantages such as ease of use, portability, and the ability to function on any surface and in various conditions [6,7,8,9]. In this context, the Enode/Vmaxpro (Blaumann and Meier-Sports Technology UG, Magdeburg, Germany) was developed—a small inertial sensor (i.e., accelerometer) that can be easily attached to metal surfaces with magnets or secured with an elastic band to a reference point on the body or an object to analyze its kinematic parameters, and information can be obtained immediately [10].

This IMU can record acceleration data across all three axes and is primarily designed for acceleration and angular velocity data, and, from those, one can determine segment kinematics [1]. Its validity has been demonstrated in free-weight tests [10,11] and vertical jumps [12]. Studies have also shown that this device is sufficiently reliable [10,13,14] and sensitive [1,15] and can be considered a valid, sensitive, and reliable instrument for velocity monitoring.

However, previous research has focused exclusively on the reliability and sensitivity of the Enode/Vmaxpro sensor during free weight exercises or jumps, leaving its applicability in isolated movements on fitness equipment unexplored. Although the logic behind its use is sound, it has not been scientifically tested. This study could be beneficial to fitness professionals, researchers, and equipment manufacturers by improving training and assessment protocols and opening up opportunities for further research, specifically in determining and understanding the sensor’s metric characteristics across different sample types and during exercises or testing on stationary fitness equipment.

Accordingly, this study aimed to define the reliability and sensitivity of the parameters measured by the Enode/Vmaxpro sensor in relation to different testing conditions, physical activity levels, and sex. We hypothesized that the Enode/Vmaxpro sensor would demonstrate good reliability and sensitivity.

## 2. Materials and Methods

This research included 27 participants, consisting of 15 men and 12 women. The entire sample was divided into physically inactive (InA) and physically active (A) participants. Among the men, 8 were inactive and 7 were active, while the women comprised 6 inactive and 6 active participants (Table 1). The physically active group consisted of individuals who regularly participated in various physical activities (fitness, running, swimming, combat sports) at least three times per week for 60–90 min of moderate to intense exercise. The physically inactive group consisted of individuals who did not engage in any systematic or planned physical activities. The exclusion criteria for participants from the study were as follows: non-healthy participants or participants who reported any pain in the form of injury or had exercised their lower extremities in the previous 48 h.

This study was approved by the Ethics Committee of the Faculty of Physical Education and Sports, University of East Sarajevo (number: 1497/23). Participants were thoroughly briefed about the tests to be conducted and informed about the study’s objectives. Only individuals who voluntarily agreed to participate and signed a written informed consent form were included in the study. The research was conducted in accordance with the Declaration of Helsinki, which provides ethical principles for medical research involving human participants [16].

### 2.1. Experimental Approach to the Problem

**Figure 1:**
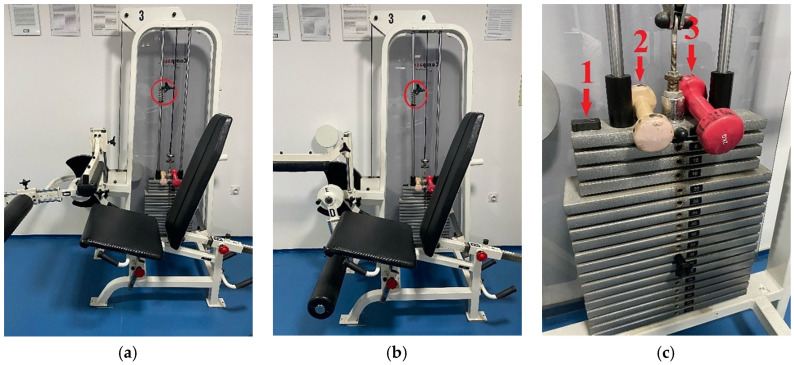
(**a**) The starting position of the machine for the flexion in knee joint. (**b**) The starting position of the machine for the extension in knee joint. (**c**) The machine load with the sensor position (1) and additional weights (2 and 3). The range of motion was secured with a stopper (red circles in Figure 1a,b).

The research was conducted using a combined leg extension/leg curl machine with a consistent weight transmission system (Figure 1. Each participant began with a general warm-up on a bicycle ergometer for 5 min, with a progressive increase in intensity each minute, followed by brief dynamic stretching of the leg muscles (quadriceps, hamstrings, posterior shank muscles, and hip flexors) for 15–30 s per stretch, with 2–4 repetitions per muscle group, ensuring the muscles remain flexible without compromising strength performance [17]. Afterward, participants proceeded to the testing machine for a specific leg warm-up, consisting of one set of 8–10 repetitions with submaximal load at varying speeds and one set of 3–5 repetitions at maximum speed for the given load [14,15], followed by another dynamic stretch for a minimum of 5 min as recommended by previous researchers [18].

Before testing, participants were secured with belts (around the chest, hips, and upper thigh of the active leg) to isolate the action of the tested muscle groups [14,15]. Extension (LE) and flexion (LC) movements of the lower leg were performed individually for each leg (right—R, left—L), with two maximum attempts per movement [14,15]. Of course, both repetitions were used to determine reliability, but they were also treated as two independent items to assess sensitivity. Each repetition was performed on the examiner’s command, with a 5–10 s break between attempts to avoid cumulative effects and allow for a return to the starting position [14,15]. Participants were instructed to extend or flex their knee to the end of the joint’s range of motion as quickly as possible. The order of flexion and extension for the right and left legs was randomized. The machine was adjusted to match each participant’s individual body dimensions.

The initial knee joint position was approximately 180° for flexion and slightly less than 90° for extension, with the final position determined by the participant’s knee joint mobility [14,15]. The range of motion, and consequently the weight movement, was defined on the machine’s guides to neutralize load inertia during the movement and ensure realistic results and was secured with a stopper (Figure 1a,b). The load was standardized at 30% of each participant’s body mass [14,15], measured using bioelectrical impedance (InBody 720, Cerritos, CA, USA) immediately before the main test. If the required weight could not be achieved using the machine’s standard weights, additional weight was added manually (Figure 1c, markers 2 and 3). The entire data collection process took one week, with each participant completing their testing in a single day.

We chose this type of protocol for two reasons: (1) the repetitive method is an integral part of strength training, so when implementing the sensor in practice, it is essential to assess its sensitivity between repetitions, and (2) the research also included inactive participants. We wanted to avoid the effects of fatigue and muscle inflammation the following day, which could potentially impact the reliability of the results for this group.

The Enode/Vmaxpro sensor, placed atop the defined load (Figure 1c, marker 1), includes a triaxial accelerometer, gyroscope, and magnetometer. This sensor had a sampling rate of 1000 Hz, weighed only 16 g, and measured 4.5 × 2.7 × 1.2 cm [10]. The device integrates the acceleration signal internally and transmits data in real time via Bluetooth (65 Hz) to a smartphone or tablet running the Vmaxpro app Ver. 1 (Blaumann and Meyer-Sports Technology UG, Magdeburg, Germany). The app allows users to view data instantly and export it in CSV format. The device was calibrated according to the manufacturer’s specifications. The repeated testing was carried out within a very short time interval, so it was not necessary to repeat the calibration. The device itself requires calibration each time it is powered up [19,20].

### 2.2. Variables

Besides the basic morphological characteristics (Table 1), the following biomechanical variables were examined: average velocity (AV) and peak velocity (PV) expressed in meters per second (m/s), average power (AP) and peak power (PP) expressed in watts (W), movement length of the Enode/Vmaxpro sensor (DIS) expressed in centimeters (cm), and duration of movement (DUR) expressed in seconds (s).

### 2.3. Statistical Analyses

All analyses were conducted using the Statistical Package for the Social Sciences (SPSS 23.0, IBM, Armonk, NY, USA). The Kolmogorov-Smirnov test showed that the data follow a normal distribution. The presented results include the mean, standard deviation (SD), and standard error (St. Err.). Reliability indicators were calculated using Pearson correlation and intraclass correlation (ICC; Two-way mixed, Average measures) [21]. Pearson’s *r* values were interpreted using the widely accepted thresholds proposed by Cohen (1988), where a small correlation is defined as 0.1–0.3, a moderate correlation as 0.3–0.5, and a large correlation as greater than 0.5 [22]. ICC values were interpreted as poor (<0.5), moderate (0.5–0.75), good (0.75–0.9), or excellent (>0.9) reliability [23]. Between-group differences were determined using multivariate analysis of variance (MANOVA). A *t*-test and Bonferroni post hoc analysis were applied to calculate differences between individual groups (measurements, right vs. left leg, leg extension vs. leg curl, physically active vs. inactive, male vs. female). The differences identified, both overall and within each group, were analyzed to determine the level of sensitivity between the studied groups. The level of significance was set at *p* < 0.05 [24].

## 3. Results

The primary data from Table 2 demonstrate that the Enode/Vmaxpro sensor exhibited high reliability across all tested variables (AV, PV, AP, PP, DIS, DUR) in both trials (Trial 1 and Trial 2). Descriptive statistics (Mean, SD, St. Err.) reveal minimal variation between the two trials, indicating consistent performance measurements. Reliability indicators, including Pearson Correlation (0.881–0.983 for ALL, 0.817–0.968 for MALE, 0.841–0.984 for FEMALE) and ICC values (0.937–0.991 for ALL, 0.899–0.984 for MALE, 0.908–0.990 for FEMALE), underscore statistically significant reliability (*p* ≤ 0.05) for all monitored variables. Furthermore, the absence of statistically significant differences in *t*-test results (*p* = 0.061–1.000) between repeated attempts supports the sensor’s consistency, making it a robust tool for assessing isolated movements on fitness equipment across diverse populations.

The sensitivity of the Enode/Vmaxpro device was assessed by analyzing the differences presented in Table 3 and Table 4. Table 3 outlines the statistically significant differences between sexes (Male/Female, Wilks’ Lambda = 0.000) and between specific subgroups individually (R-L; LE-LC; InA-A), as well as their combined interactions (R-L/LE-LC; InA-A/LE-LC; InA-A/LE-LC/R-L).

An analysis of the relationships between the examined subgroups revealed no statistically significant differences between the right and left legs overall (M: *p* = 0.388; F: *p* = 0.565), nor when observing each leg’s performance individually as a function of movement (LE-M: *p* = 0.405, F: *p* = 0.679; LC-M: *p* = 0.808, F: *p* = 0.462) (Table 3).

Significant differences were found when comparing movement types (LE-LC) and activity levels (InA-A) regardless of sex (*p* = 0.000). When examining activity levels as a function of movement, significant differences were observed for men (*p* = 0.000) and for women, with *p* values ranging from 0.000 to 0.011.

However, the true level of sensitivity is best determined by analyzing the differences in specific movements (LE or LC) for the right or left leg as a function of activity level and sex (M: *p* = 0.001–0.017; F: *p* = 0.011–0.045), as shown in Table 3.

In addition to the general differences between sexes and the observed differences within the examined subgroups (Table 3), Table 4 presents the descriptive results and analysis of individual variable differences across the defined subgroups in their overall interactions (M-F/InA-A/LE-LC/R-L). These findings highlight significant differences between physically active and inactive participants across variables such as movement type (dominant vs. non-dominant legs), leg dominance, and sex.

Table 4 outlines that active participants consistently showed higher AV, PV, AP, and PP compared to inactive participants. The differences were more pronounced for men, especially in dominant legs (e.g., PP in dominant right leg for males: F = 0.020, Sig. ≤ 0.05). For women, power-related metrics such as AP and PP also demonstrated significant differences, particularly in dominant legs (F ≤ 0.025).

Inactive participants exhibited longer DIS in some cases, but these differences are less consistent across groups and sexes. Significant results are observed for males in specific dominant leg conditions (F = 0.046, Sig. ≤ 0.05).

Active individuals demonstrated shorter DUR, indicating faster execution of tasks compared to inactive counterparts. Significant differences were observed across both sexes and leg dominance (e.g., F = 0.001–0.008 for males in dominant and non-dominant legs).

Analyzing the results of this research, we can confidently state that all examined parameters were reliable, both generally and in relation to sex. Notably, the reliability of the analyzed parameters in women was slightly higher than in men, both overall (Male: ICC = 0.956; Female: ICC = 0.963) and individually across almost all variables (DUR, AV, PV, AP, PP). It should also be noted that AV and AP had higher reliability than PV and PP, suggesting that the average speed and power parameters might be a better choice. However, the high reliability of PV and PP also allows for their use if they are of diagnostic interest.

## 4. Discussion

Despite the manufacturer’s claim that the Enode/Vmaxpro sensor is valid, reliable, and sensitive, several studies have addressed the metric characteristics of this sensor in recent years [10,12,14,20,25]. Although previous studies have examined the reliability of this device, none focused on its application to fitness equipment. This study fills that gap by evaluating the reliability and sensitivity of the Enode/Vmaxpro sensor when used with such equipment, expanding on our previous research, which was based on a different and smaller sample and focused on free-weight strength training.

Previous research has confirmed the sufficient level of reliability of the Enode/Vmaxpro in exercise and testing conditions with free weights (e.g., squats and hip thrusts) both within and between days [10]. It has also shown reliability during countermovement jumps (CMJs), with the sensor’s position on the ankle joint being more reliable than on the hip [13]. Additionally, our previous research established the reliability of this sensor on a combined leg curl and leg extension device, both overall and individually during flexion and extension [14].

When comparing the reliability results of this study with previous research [10,13,14], it is evident that the determined values are either identical to or higher than those previously found, which is expected given that this study was conducted on a single-joint exercise machine.

Previous research on sensitivity analyzed this metric characteristic and confirmed a sufficient level of sensitivity for diagnosing minimal changes in speed during squat exercises and when increasing external load [1]. This research focused on sensitivity in response to various external loads, while we sought to expand this by examining sensitivity in all relationships. Marković and associates [15] initiated research on sensitivity in terms of training, movement type, and leg dominance, and this study builds on that work, expanding it with a larger sample and including sex as a factor.

At first glance, patterns related to movement type and sex can be observed, while leg dominance appears to have no significant effect. Regarding leg extension (LE), a statistically significant sensitivity was observed in men for the variables PP, DIS, and DUR for both the right (*p* = 0.000–0.048) and left legs (*p* = 0.001–0.046), with DUR being the most sensitive variable for both legs (*p* = 0.000 and *p* = 0.001, respectively), favoring physically active participants. In women, during the same movement, significant differences were found in the variables AP, PP, and DUR for both the right (*p* = 0.002–0.025) and left legs (*p* = 0.001–0.002), again favoring physically active participants.

During the leg curl (LC) movement, men showed statistically significant differences in all monitored variables, regardless of leg (R: *p* = 0.001–0.048; L: *p* = 0.001–0.020), except for the variable DIS for the right leg, where no sufficient level of sensitivity was found (*p* = 0.514). In women, the most sensitive parameters during the same movement were AP and PP, with AP being statistically significant for the right leg (*p* = 0.026) and PP approaching significance (*p* = 0.080), and for the left leg, AP approaching significance (*p* = 0.070) and PP being statistically significant (*p* = 0.038). These observed patterns emphasize the need to examine all metric characteristics, especially sensitivity, in relation to various test factors, such as the participants’ training, sport, testing conditions, movement type, and muscle groups. Of course, all sensitivity levels that are on the borderline of significance will need to be re-examined in future research with a larger number of participants.

In general, the PP variable achieved a statistically significant difference in nearly all observed relationships between active and inactive participants, except for women during the LC movement with the right leg. Additionally, for men, the DUR variable showed high sensitivity regardless of movement type or dominance, while for women, this variable was more sensitive during the LE movement. Regardless of the determined level of differences, descriptive results indicate higher values for AV, PV, AP, and PP in favor of active participants, regardless of sex, movement type, or dominance. Interestingly, the DUR variable showed lower values in active participants, except in the case of women during the LC movement of the left leg. Moreover, during the LE movement, the DIS variable had lower values in active participants, regardless of sex, while during the LC movement, the length of movement was generally longer for active participants. This further highlights the specificity of the movement and the need to define all levels of sensitivity.

The main limitation of this study is the small sample size, as well as the absence of groups with different training regimes (e.g., football, basketball, handball). Nonetheless, this study serves as a foundation for future research, with these limitations suggesting directions for further investigation.

## 5. Conclusions

Following the aim of this research, we can conclude that all the measured parameters (AV, PV, AP, PP, DIS, DUR) are reliable, both in general and as a function of sex. Therefore, we can say that the Enode/Vmaxpro sensor is reliable for performance testing, both in terms of length of movement, duration of movement, speed of movement, and also muscular strength.

Regarding the sensitivity, we conclude that the examined parameters were sensitive when we compare the obtained results by sex (male and female) but also by type of movement (LC and LE) and activity level (InA and A) also in function of sex. However, in order to improve the training process itself, it was necessary to determine the sensitivity levels of all examined parameters as a function of sex and activity level when performing both flexion and extension with the right and left leg individually. In this regard, the sensitivity of the examined variables in men during LE movement was determined in PP, DIS, and DUR during LC movement in all variables in general, while in women a significant level of sensitivity during LE was determined in AP, PP, DUR, and at LC in AP and PP. Therefore, we conclude that the Enode/Vmaxpro sensor is also sensitive enough to evaluate the manifested performance through the tested biomechanical variables, and we can highlight PP as one of the most sensitive.

## Figures and Tables

**Table 1:** Basic descriptive indicators of the participants (mean ± SD).

Sex	Group	N	Age	BH (cm)	BM (kg)	BMI (kg/m^2^)	PBFM (%)	PSMM (%)
Male	All	15	33.0 ± 9.6	181.6 ± 7.1	85.6 ± 14.2	25.9 ± 3.4	17.4 ± 6.1	47.3 ± 3.7
	InA	8	34.1 ± 4.9	182.7 ± 8.7	84.7 ± 15.6	25.2 ± 3.3	20.9 ± 5.1	45.0 ± 2.8
	A	7	32.1 ± 12.4	180.7 ± 6.0	86.3 ± 13.9	26.4 ± 3.6	14.7 ± 5.7	49.1 ± 3.3
Female	All	12	32.2 ± 2.7	168.6 ± 7.1	68.9 ± 7.2	24.1 ± 4.0	28.5 ± 6.8	39.4 ± 3.7
	InA	6	33.5 ± 2.1	166.0 ± 5.7	70.6 ± 8.7	25.4 ± 7.5	30.9 ± 9.6	37.8 ± 4.5
	A	6	31.3 ± 3.1	170.3 ± 8.5	67.7 ± 5.6	23.3 ± 0.9	26.9 ± 6.1	40.5 ± 3.6

Legend: InA—Physically Inactive Participants; A—Physically Active Participants; BH—Body Height in cm; BM—Body Mass in kg; BMI—Body Mass Index in kg/m^2^; PBFM—Percent of Body Fat Mass; Percent of Skeletal Muscle Mass.

**Table 2:** Descriptive statistics, reliability metrics, and comparative analysis of performance variables.

		Trial	Mean	SD	St. Err.	Correlation	ICC	*t*
ALL	AV (m/s)	I	0.672	0.119	0.013	0.969 *	0.984 *	−1.876
		II	0.678	0.118	0.013			
	PV (m/s)	I	1.040	0.224	0.024	0.956 *	0.977 *	−1.716
		II	1.053	0.226	0.025			
	AP (W)	I	179.202	49.410	5.391	0.983 *	0.991 *	−2.071
		II	181.298	50.319	5.490			
	PP (W)	I	282.619	95.202	10.387	0.973 *	0.986 *	−2.212
		II	288.107	98.527	10.750			
	DIS (cm)	I	44.817	5.718	0.624	0.881 *	0.937 *	−0.827
		II	45.065	5.593	0.610			
	DUR (s)	I	0.679	0.088	0.010	0.886 *	0.940 *	0.749
		II	0.675	0.089	0.010			
MALE	AV (m/s)	I	0.707	0.092	0.012	0.944 *	0.971 *	−1.487
		II	0.713	0.094	0.012			
	PV (m/s)	I	1.102	0.179	0.022	0.930 *	0.963 *	−1.909
		II	1.118	0.186	0.023			
	AP (W)	I	197.266	38.942	4.868	0.968 *	0.984 *	−1.678
		II	199.375	40.339	5.042			
	PP (W)	I	314.719	80.740	10.092	0.956 *	0.977 *	−2.248
		II	321.672	83.982	10.498			
	DIS (cm)	I	45.686	5.242	0.655	0.886 *	0.939 *	0.078
		II	45.661	5.438	0.680			
	DUR (s)	I	0.651	0.058	0.007	0.817 *	0.899 *	1.546
		II	0.644	0.060	0.007			
FEMALE	AV (m/s)	I	0.561	0.131	0.029	0.984 *	0.990 *	−1.245
		II	0.568	0.120	0.027			
	PV (m/s)	I	0.843	0.242	0.054	0.971 *	0.983 *	0.000
		II	0.843	0.219	0.049			
	AP (W)	I	121.400	31.946	7.143	0.981 *	0.990 *	−1.445
		II	123.450	32.279	7.218			
	PP (W)	I	179.900	58.312	13.039	0.971 *	0.985 *	−0.255
		II	180.700	55.520	12.415			
	DIS (cm)	I	42.035	6.402	1.432	0.862 *	0.924 *	−1.547
		II	43.160	5.792	1.295			
	DUR (s)	I	0.768	0.108	0.024	0.841 *	0.908 *	−0.573
		II	0.776	0.094	0.021			

Legend: AV—Average Velocity and PV—Peak Velocity in m/s; AP—Average Power and PP—Peak Power in W; DIS—Length of Movement in cm; DUR—Duration of Movement in s; SD—Standard Deviation; St. Err.—Standard Error; ICC—Intraclass Correlation Coefficient; *t*—*t*-Statistic Values; * Significant at *p* ≤ 0.05.

**Table 3:** Multivariate analysis of variance (MANOVA) between- and within-group results.

Effect				Value	F	Hypothesis df	Error df	Sig.
Between the sexes Male/Female			Wilks’ Lambda	0.493	27.625	6.000	161.000	0.000 *
**Differences within sexes**			**Male**			**Female**		
Sub Groups			**Value**	**F**	**Sig.**	**Value**	**F**	**Sig.**
R-L			0.95	1.065	0.388	0.871	0.816	0.565
LE-LC			0.361	35.687	0.000 *	0.323	11.543	0.000 *
R-L		LE	0.901	1.048	0.405	0.765	0.666	0.679
		LC	0.95	0.497	0.808	0.683	1.005	0.462
InA-A			0.709	8.294	0.000 *	0.293	13.288	0.000 *
InA-A		LE	0.572	7.117	0.000 *	0.057	35.647	0.000 *
		LC	0.597	6.423	0.000 *	0.323	4.548	0.011 *
InA-A	LE	R	0.548	3.443	0.013 *	0.022	22.163	0.014
		L	0.441	5.275	0.001 *	0.032	15.111	0.024 *
	LC	R	0.501	4.151	0.005 *	0.049	9.658	0.045 *
		L	0.562	3.243	0.017 *	0.019	25.735	0.011 *

Legend: R—Right Leg; L—Left Leg; LE—Leg Extension; LC—Leg Curl; InA—Physically Inactive Participants; A—Physically Active Participants; F—F-Statistic Value; df—Degrees of Freedom; Sig.—Significance (* Significant at ≤0.05).

**Table 4:** Descriptive statistics and analysis of differences between physically active and inactive individuals regarding movement type, limb dominance, and sex.

Group		Sex		Male					Female				
		Variables	Inactive-Active	Mean	SD	St. Err.	F	Sig.	Mean	SD	St. Err.	F	Sig.
LE	R	AV (m/s)	InA	0.759	0.079	0.021	0.507	0.229	0.615	0.007	0.005	4.673	0.523
			A	0.788	0.059	0.014			0.676	0.124	0.044		
		PV (m/s)	InA	1.201	0.136	0.036	1.507	0.130	0.875	0.007	0.005	3.035	0.422
			A	1.263	0.089	0.021			1.034	0.254	0.090		
		AP (W)	InA	209.143	32.415	8.663	0.070	0.162	103.500	2.121	1.500	6.043	0.007 *
			A	226.278	34.435	8.116			156.125	19.924	7.044		
		PP (W)	InA	337.429	59.714	15.959	0.089	0.020 *	145.500	3.536	2.500	76.613	0.025 *
			A	391.556	63.512	14.970			237.125	44.908	15.877		
		DIS (cm)	InA	51.207	6.807	1.819	3.296	0.048 *	53.500	1.414	1.000	3.262	0.110
			A	47.111	4.424	1.043			45.700	5.835	2.063		
		DUR (s)	InA	0.674	0.047	0.012	1.727	0.000 *	0.875	0.035	0.025	0.301	0.002 *
			A	0.598	0.060	0.014			0.684	0.056	0.020		
	L	AV (m/s)	InA	0.766	0.084	0.023	0.305	0.697	0.595	0.021	0.015	2.175	0.360
			A	0.776	0.057	0.014			0.660	0.090	0.032		
		PV (m/s)	InA	1.212	0.156	0.042	0.032	0.123	0.875	0.021	0.015	1.752	0.240
			A	1.292	0.127	0.030			1.041	0.177	0.063		
		AP (W)	InA	210.857	39.463	10.547	1.217	0.352	100.500	3.536	2.500	1.457	0.001 *
			A	223.111	33.870	7.983			152.875	12.688	4.486		
		PP (W)	InA	344.714	69.452	18.562	0.021	0.039 *	146.000	2.828	2.000	3.476	0.001 *
			A	401.944	77.877	18.356			238.750	25.617	9.057		
		DIS (cm)	InA	50.764	6.454	1.725	2.975	0.046 *	50.700	0.000	0.000	3.940	0.180
			A	46.850	4.157	0.980			45.125	5.126	1.812		
		DUR (s)	InA	0.664	0.039	0.010	2.001	0.001 *	0.850	0.028	0.020	1.310	0.002 *
			A	0.603	0.053	0.013			0.686	0.048	0.017		
LC	R	AV (m/s)	InA	0.612	0.077	0.021	7.543	0.010 *	0.455	0.007	0.005	2.969	0.821
			A	0.671	0.044	0.010			0.464	0.050	0.018		
		PV (m/s)	InA	0.906	0.118	0.032	3.288	0.004 *	0.655	0.007	0.005	3.879	0.986
			A	1.014	0.082	0.019			0.658	0.091	0.032		
		AP (W)	InA	164.714	34.965	9.345	0.749	0.048 *	74.500	0.707	0.500	2.275	0.026 *
			A	187.944	28.829	6.795			104.250	14.714	5.202		
		PP (W)	InA	239.000	48.181	12.877	1.796	0.014 *	107.500	0.707	0.500	2.602	0.080
			A	280.111	40.717	9.597			142.625	23.688	8.375		
		DIS (cm)	InA	42.550	2.686	0.718	3.668	0.514	38.850	0.212	0.150	4.851	0.959
			A	43.039	1.454	0.343			38.738	2.837	1.003		
		DUR (s)	InA	0.703	0.062	0.017	5.566	0.001 *	0.860	0.014	0.010	10.101	0.750
			A	0.643	0.030	0.007			0.840	0.082	0.029		
	L	AV (m/s)	InA	0.604	0.048	0.013	1.138	0.001 *	0.465	0.007	0.005	2.113	0.734
			A	0.682	0.063	0.015			0.490	0.096	0.034		
		PV (m/s)	InA	0.912	0.082	0.022	1.003	0.001 *	0.665	0.021	0.015	2.173	0.620
			A	1.032	0.097	0.023			0.719	0.141	0.050		
		AP (W)	InA	163.357	30.938	8.269	0.050	0.019	77.500	0.707	0.500	3.950	0.070
			A	191.111	31.991	7.540			109.875	20.966	7.412		
		PP (W)	InA	242.000	45.706	12.216	0.154	0.015 *	106.500	2.121	1.500	4.009	0.038 *
			A	284.444	46.316	10.917			156.625	27.370	9.677		
		DIS (cm)	InA	41.136	2.832	0.757	0.416	0.020 *	36.000	4.384	3.100	0.144	0.352
			A	43.389	2.339	0.551			38.663	3.246	1.148		
		DUR (s)	InA	0.684	0.040	0.011	0.442	0.008 *	0.770	0.085	0.060	0.656	0.633
			A	0.639	0.047	0.011			0.810	0.104	0.037		

Legend: AV—Average Velocity and PV—Peak Velocity in m/s; AP—Average Power and PP—Peak Power in W; DIS—Length of Movement in cm; DUR—Duration of Movement in s; SD—Standard Deviation; St. Err.—Standard Error; F—F-Statistic Value; Sig.—Significance (* Significant at ≤0.05).

## Data Availability

The data provided in this study can be obtained upon request from the corresponding author.
